# Determination of Antibacterial and Technological Properties of Vaginal Lactobacilli for Their Potential Application in Dairy Products

**DOI:** 10.3389/fmicb.2017.00166

**Published:** 2017-02-07

**Authors:** Lorenzo Siroli, Francesca Patrignani, Diana I. Serrazanetti, Carola Parolin, Rogers A. Ñahui Palomino, Beatrice Vitali, Rosalba Lanciotti

**Affiliations:** ^1^Department of Agricultural and Food Sciences, Alma Mater Studiorum, University of BolognaCesena, Italy; ^2^Interdepartmental Center for Industrial Agri-Food Research, University of BolognaCesena, Italy; ^3^Department of Pharmacy and Biotechnology, University of BolognaBologna, Italy

**Keywords:** vaginal lactobacilli, functional foods, adjunct cultures, antibacterial activity, technological properties

## Abstract

Functional foods could differently affect human health in relation to the gender. Recent studies have highlighted the anti-*Candida* and anti-*Chlamydia* activities of some *Lactobacillus* strains isolated from the vagina of healthy women. Considering these important beneficial activities on women's health, the preparation of functional food containing active vaginal lactobacilli can represent a great scientific challenge for the female gender. In this context, the aim of this work was to study some functional and technological properties of 17 vaginal strains belonging to the species *Lactobacillus crispatus, Lactobacillus gasseri*, and *Lactobacillus vaginalis* in the perspective to include them in dairy products. The antagonistic activities against the pathogenic and spoilage species associated to food products and against the principal etiological agents of the genitourinary tract infections were evaluated. Moreover, the vaginal lactobacilli were characterized for their antibiotic resistance, and for their fermentation kinetics and viability during the refrigerated storage in milk. Finally, the volatile molecule profiles of the obtained fermented milks were determined. The results showed that several strains, mainly belonging to the species *Lactobacillus crispatus*, exhibited a significant antagonistic activity against spoilage and pathogenic microorganisms of food interest, as well as against urogenital pathogens. All the vaginal lactobacilli showed antimicrobial activity against strains belonging to the foodborne pathogenic species *Listeria monocytogenes, Listeria innocua, Eenterococcus faecalis* and *Escherichia coli*. In addition, most of the *Lactobacillus* strains were active toward the main pathogens responsible of vaginal and urinary tract infections including *Staphylococcus aureus, Enterococcus faecium, Gardnerella vaginalis*, and *Proteus mirabilis*. The antimicrobial activity can be attributed to the high production of organic acids. The fermentation kinetics in milk indicated the unsuitability of these lactobacilli as fermentation starters for the industrial production of dairy products. However, some strains, belonging to the species *Lactobacillus crispatus* and *Lactobacillus gasseri*, maintained a high viability in pasteurized milk at 4°C for over a month, showing their potential application as adjunct cultures for the production of female gender foods. These data represent a first step for the set-up of a new functional dairy product, directed to the women well-being, contributing also to innovate the dairy sector.

## Introduction

Functional food is currently one of the fastest growing sector of food industry because considers a dietary strategy to reduce incidence of human illness (Gatti et al., 2004; Granato et al., 2010; Lobo et al., 2010; Burns et al., 2015). The literature data clearly indicate that functional foods could differently affect human health in relation to the gender. In recent decades, research on the female gender has been neglected, and the results obtained in men have been directly translated to women both in medicine and nutrition fields (Marino et al., 2011). The European Commission's Concerted Action on Functional Food Science in Europe (FuFoSE), coordinated by the International Life Science Institute (ILSI), defines functional foods as “products having beneficial effects on one or more functions of the human organism thus either improving the general and physical conditions or/and decreasing the risk of the evolution of diseases.” Moreover, this Institution states that “the amount and form of functional foods must be compatible with the nutritional parameters of a proper diet for the human organism.” Therefore, probiotic foods have received a lot of attention, because ingestion of probiotic cultures exerts beneficial effects on the gastrointestinal and urogenital tracts (Barrons and Tassone, 2008; Ceapa et al., 2013).

Reproductive-aged women are often subjected to gynecological disorders caused by vaginal microbiota dysbiosis and the possible occurrence of vaginal infections, including vulvovaginal candidiasis, bacterial vaginosis, aerobic vaginitis and sexually transmitted diseases (Workowski and Berman, 2010; Di Vito et al., 2015). Vulvovaginal candidiasis is a pathogenic yeast infections (30–35% caused by *Candida albicans*) that compromise the quality of life of women (das Neves et al., 2008). Bacterial vaginosis is generally caused by an imbalance in the ecology of the normal vaginal microbiota, characterized by a decrease of *Lactobacillus* species, which predominate in the vaginal microbiota of healthy women, and an increase in the prevalence of anaerobic pathogenic bacteria (van de Wijgert et al., 2014; Green et al., 2015; Machado and Cerca, 2015). Aerobic vaginitis represents another of the major dismicrobisms of the vaginal microbiota where lactobacilli are replaced with aerobic organisms such as streptococci, enterococci, *Escherichia coli* and *Staphylococcus aureus* (Han et al., 2014). Bacterial vaginosis and aerobic vaginitis may negatively interfere with women's reproductive health, increasing the risk of sexually transmitted infections, risk of abortion, preterm delivery, premature rupture of membranes and chorioamnionitis (Geng et al., 2016; Redelinghuys et al., 2016). Conventional therapies used to treat these disorders are generally ineffective and cause frequent relapses. On the contrary, the administration of probiotics has resulted in a very effective strategy to restore the normal vaginal microbiota (Borges et al., 2014). Beyond the vaginal application, probiotic bacteria can be administered orally thanks to their ability to pass from the intestine to the vagina, with a consequent beneficial impact on the vaginal habitat, as demonstrated by several investigations (Reid et al., 2001; Strus et al., 2012; Vitali et al., 2012; Vujic et al., 2013; Heczko et al., 2015).

Recent studies have highlighted the anti-*Candida* (Parolin et al., 2015) and anti-*Chlamydia* (Nardini et al., 2016) activities of some *Lactobacillus* strains isolated from the vagina of healthy women. Considering these important beneficial activities on women's health, the preparation of functional food containing active vaginal lactobacilli can represent a great scientific challenge for the female gender. A wide literature indicates that dairy products, such as fermented milks and cheese, represent excellent carriers of probiotic bacteria, because they are characterized by a high buffer capacity and a protective lipoprotein matrix. These properties allow to better protect the microbial cells during the gastrointestinal transit (Gomes da Cruz et al., 2009; Lollo et al., 2013; Castro et al., 2015). Moreover, dairy products, such as cheese and fermented milks, are perceived positively by consumers because they represent a good source of calcium, phosphorus and vitamins; therefore, the use of a cheese or a fermented milk can be a strategy for the ingestion of probiotic strains capable of positively influencing the healthy status of the human vaginal microbiota, in order to protect the woman from vaginal infections.

In this context, the aim of this work was to evaluate the amensalistic properties and the technological potential of 17 vaginal strains belonging to the species *Lactobacillus crispatus, Lactobacillus gasseri*, and *Lactobacillus vaginalis*, in the perspective to include them in dairy products. In particular, these lactobacilli were evaluated for their antagonistic activity against the pathogenic and spoilage species frequently associated to food products and against the principal etiological agents of bacterial vaginosis, aerobic vaginitis and urinary tract infections. Moreover, the 17 strains were characterized for their bacteriocin production and antibiotic susceptibility. Finally, the fermentation kinetics and viability of these lactobacilli during the refrigerated storage in milk were characterized, as well as the volatile molecule profiles of the obtained fermented milks.

## Materials and methods

### Microbial strains and growth conditions

Seventeen *Lactobacillus* (*L*.) strains were considered in this work (Collection of the Department of Pharmacy and Biotechnology of Bologna University). These lactobacilli were previously isolated from vaginal swabs obtained from pre-menopausal Caucasian women (aged 18–45 years old), who have no symptoms of vaginal or urinary tract infection (Ethics Committee of the University of Bologna: 52/2014/U/Tess) (Parolin et al., 2015). They belong to three species highly represented in the vaginal habitat: *L. crispatus* (BC1-BC8), *L. gasseri* (BC9-BC14) and *L. vaginalis* (BC15-BC17). The strains *Listeria monocytogenes* ATCC 13932 and Scott A, *Listeria innocua* DSM 20649, *Enterococcus faecalis* ATCC 29212, EF37, and BC101, *Enterococcus faecium* BC104, *Escherichia coli* DSM 18039, 555, and ATCC 11105, *Staphylococcus aureus* ATCC 29213 and DSM 20231, *Lactobacillus plantarum* V7B3, *Bacillus cereus* ATCC 11966, *Salmonella enteritidis* E5, *Enterococcus faecium* BC105, *Gardnerella vaginalis* BC106, and *Proteus mirabilis* ATCC 29906, used as target microorganisms for the determination of antagonistic activity, belong to the Department of Agricultural and Food Sciences of Bologna University or to the Department of Pharmacy and Biotechnology of Bologna University.

*L. gasseri, L. crispatus*, and *L. vaginalis* strains were grown in de Man, Rogosa and Sharpe (MRS) broth (Oxoid Ltd., Basingstoke, UK) at 37°C for 24 h in anaerobic conditions using 3.5 liter anaerobic jars with AnaeroGen paper sachets (Oxoid Ltd., Basingstoke, UK). Pathogenic and spoilage strains were cultured in Brain Heart Infusion (BHI) broth (Oxoid Ltd.) at 37°C for 24 h.

### Evaluation of antagonistic activity and bacteriocin production of vaginal lactobacilli

To evaluate the ability of the vaginal lactobacilli to antagonize the target strains reported in Section Microbial Strains and Growth Conditions, the method of Schillinger and Lücke (1989) was applied with some modifications. Five μl of the overnight cultures of the vaginal lactobacilli were poured over the surface of MRS plates (containing 1.2% of agar) and allowed to grow under anaerobic conditions at 37°C for 24 h. Then 0.1 mL (corresponding to approximately 7 log CFU) of an overnight culture of the target strain was inoculated into 10 mL of BHI soft agar (containing 0.7% of agar) and poured on the plates where the vaginal lactobacilli had grown. After an incubation at 37°C for 24 h, the plates were checked to evaluate the zone of growth inhibition. The inhibition halos were measured from the outer perimeter of the spots in four directions and the average diameters was considered. The antagonistic activity was expressed in relation to the observed diameter of inhibition: −, No inhibition; +, Diameter between 1 and 3 mm; ++, Diameter between 3 and 6 mm; +++, Diameter between 6 and 10 mm; ++++, Diameter > 10 mm.

For the evaluation of bacteriocins production the method reported by Schillinger and Lücke (1989) with some modification was used, 24 h cultures of the vaginal lactobacilli were centrifuged (4000 rpm for 5 min) and the supernatants were heated at 98°C for 5 min or filtered with 0.20 μm cellulose acetate filters. The cell-free supernatants were neutralized to pH 6.5 by the addition of 1N NaOH. Then, 10 mL of BHI soft agar inoculated with 0.1 mL of an overnight culture of the indicator strain (*L. monocytogenes* ATCC 13932, *L. innocua* DSM 20649, *E. coli* DSM 18039 and *S. aureus* DSM 20231), were poured into petri plates. After plates drying, 5 μl of a cell-free *Lactobacillus* supernatant were poured on the plate surface. The plates were incubated at 37°C for 24 h and subsequently examined to evaluate the diameter of growth inhibition diameter.

### Antibiotic susceptibility

The antibiotic susceptibility of the vaginal lactobacilli was determined using M.I.C.E. evaluator strips (Oxoid Ltd., Basingstoke, UK). The OD_600_ of 24 h lactobacilli cultures were adjusted at 0.6, 100 uL of the cell culture (approximately 7 log CFU/mL) were inoculated on MRS agar plates and streaked over the entire surface of the plates. The inoculated plates were dried for about 15 min and finally the M.I.C.E evaluators strips were placed under sterile conditions at the center of the plates. The plates were then incubated under anaerobic conditions at 37°C for 24 h and the results were read as reported in Thermo Scientific™ Oxoid™ M.I.C.Evaluator™ (M.I.C.E.™) Strips Interpretation Guide. As control strain the *E. faecalis* ATCC29212 was used. The tested antibiotics and the relative ranges of concentrations are the followings: Amoxycillin, 256-0.015 μg/mL; Ampicillin, 256-0.015 μg/mL; Ciprofloxacin, 32-0.002 μg/mL; Clindamycin, 256-0.015 μg/mL; Erythromycin, 256-0.015 μg/mL; Gentamicin, 256-0.015 μg/mL; Levofloxacin, 32-0.002 μg/mL; Penicillin, G 32-0.002 μg/mL; Tetracycline, 256-0.015 μg/mL and Vancomycin 256-0.015 μg/mL.

### Fermentation kinetics of vaginal lactobacilli in pasteurized milk and viability at refrigerated storage

Overnight cultures of lactobacilli were inoculated at a concentration of 6 to 7 log CFU/mL in 50 mL of pasteurized whole milk, previously heated at 105°C for 7 min. The inoculated milk was incubated at 37°C and the acidification kinetics were assessed using a pH-meter basic 20 (CRIASON, Modena, Italy). The growth of each strain in fermented milk was evaluated by plate counting on MRS agar plates. The same protocol was used to evaluate the acidification kinetics in milk enriched with 0.5% tryptone after pasteurization. To assess the viability of lactobacilli in pasteurized milk in refrigeration conditions, the inoculated milk was incubated at 4°C. The viability of each strain was evaluated by plate counting on MRS agar plates. Each experiment was performed in triplicate.

### Analysis of aromatic profile in fermented milk

The flavor profiles of the vaginal lactobacilli were evaluated after growth in pasteurized milk enriched with 0.5% tryptone after pasteurization. The fermented milks were collected after 48 h of incubation at 37°C. The inoculum level and growth conditions were the same reported in Section Fermentation Kinetics of Vaginal Lactobacilli in Pasteurized Milk and Viability at Refrigerated Storage. The volatile molecule profile of the samples was determined by means of solid phase microextraction technique combined with gas chromatography and mass spectrometry (GC-MS/SPME), according to the method proposed by Burns et al. (2015). The compounds were identified by using the Agilent HewlettePackard NIST 98 mass spectral database.

## Results

### Antagonistic activity of vaginal lactobacilli

The antagonist activities of 17 vaginal strains belonging to the species *L. crispatus, L. gasseri*, and *L. vaginalis* against pathogens and spoilage microorganisms of food interest were evaluated. All tested lactobacilli showed a remarkable inhibitory activity against *L. monocytogenes* Scott A, *L. innocua* DSM 20649, *E. faecalis* EF37 and *E. coli* DSM 18039 and 555 (Table 1). In general, *L. crispatus* BC1, BC4, BC5, BC6, BC7, and BC8 showed the highest antagonistic activity. In fact, the majority of these strains determined inhibition zones ranging between 6 and 10 mm toward *L. monocytogenes* Scott A, *L. innocua* DSM 20649, *E. coli* DSM 18039, and 555. Moreover, they showed a good antagonist activity, with inhibition zones ranging between 1 and 6 mm, against *L. monocytogenes* ATCC 13932, *E. faecalis* ATCC 29212 and EF37, *S. aureus* DSM 20231, *L. plantarum* V7B3, *B. cereus* ATCC 11966, and, although at a lesser extent, against *S. enteritidis* E5. *L. crispatus* BC2 and BC3 and *L. gasseri* BC9, BC10, BC11, BC12 and BC13 showed a good antagonistic activity against *L. monocytogenes* ATCC 13932 and Scott A, *L. innocua* DSM 20649, *E. faecalis* ATCC 29212 and EF37, *E. coli* DSM 18039 and 555, *S. aureus* DSM 20231. *L. gasseri* BC14, *L. vaginalis* BC15, BC16, and BC17 showed the lowest antagonistic activity against all the target microorganisms considered.

**Table 1:** **Evaluation of the antagonist activity of the vaginal lactobacilli against several spoilage or pathogenic microorganisms associated to food products**.

		***L. monocytogenes***	***L. monocytogenes***	***L. innocua***	***E. faecalis***	***E. faecalis***	***E. faecalis***	***E. faecium***	***E. coli***	***E. coli***	***E. coli***	***S. aureus***	***S. aureus***	***L. plantarum***	***B. cereus***	***S. enteritidis***	***E. faecium***	***G. vaginalis***	***P. mirabilis***
	**Strain**	**ATCC 13932**	**SCOTT A**	**DSM 20649**	**ATCC 29212**	**EF37**	**BC101**	**BC 104**	**DSM 18039**	**555**	**ATCC 11105**	**ATCC 29213**	**DSM 20231**	**V7B3**	**ATCC11966**	**E5**	**BC 105**	**BC106**	**ATCC 29906**
*L. crispatus*	BC1	+++	+++	+++	++	+++	+++	+++	++++	+++	+++	+++	+++	+	++	+	+++	+++	++++
	BC2	+	+++	++	+	++	++	+	++	+++	+	++	+	+	+	+	+	++	++++
	BC3	+	++	+	+	+	+	+++	+	++	++	++	+	−	−	−	+	++	+++
	BC4	++	+++	+++	++	++	++	++	+++	+++	++	+	++	+	++	+	++	+++	+++
	BC5	++	++	+++	++	++	+++	+	++++	+++	++++	+++	++	++	++	+	+++	+	+++
	BC6	+	+++	+++	++	+++	+++	++	+++	+++	++++	+++	+	+	+	+	+	+	+++
	BC7	+	+++	+++	+	++	+++	+++	++	+++	++	+++	+	+	++	+	++	++	++++
	BC8	++	++	+++	+	++	+++	++	++++	++	++++	++	+	−	+++	+	+	++	+
*L. gasseri*	BC9	+	+	+	+	+	++	++	++	++	++	++	+	−	−	+	+	++	+++
	BC10	+	++	++	+	+	+	+	++	++	+++	+	++	+	+	−	++	+	++
	BC11	++	+++	+++	+	+	+	++	++	++	++	+	+	−	+	+	+	+	−
	BC12	++	++	+++	+	+	+	++	++	+++	−	+	+	−	+	+	+	+	−
	BC13	++	+	++	+	++	++	++++	+++	++	+++	++	+	+	++	+	++	+	+++
	BC14	+	+	+	−	+	+++	++	+	+	+++	+++	-	−	−	−	++++	+	+++
*L. vaginalis*	BC15	+	+	+	−	++	+++	++++	+	++	++++	+++	+	−	−	+	+++	+++	++++
	BC16	+	+	+	+	+	−	+	+	+	+	−	+	−	+	−	+	−	++
	BC17	+	+	+	+	+	+	++	+	+	+++	++	+	−	+	−	++	+	−

*Legend: −, no inhibition; +, inhibition 1–3 mm; ++, inhibition 3–6 mm; +++, inhibition 6–10 mm; ++++, >10 mm*.

*The diameter of inhibition, for each strain, was the average of three replicates*.

The majority of the vaginal lactobacilli showed antibacterial activity also toward the main pathogens responsible of vaginal and urinary tract infections (*E. faecalis* BC101, *E. faecium* BC104, *E. coli* ATCC 11105, *S. aureus* ATCC 29213, *E. faecium* BC105, *G. vaginalis* BC106, and *P. mirabilis* ATCC 29906). In particular, *L. crispatus* BC1 and *L. vaginalis* BC15 were the most active strains as their inhibition zones were greater than 6 mm for all the test pathogens. Otherwise, the lowest inhibitory activity was elicited by *L. gasseri* BC11 and BC12 and *L. vaginalis* BC16 which gave zones of inhibition lower than 6 mm and, in some cases, were not active (Table 1).

None of the considered *Lactobacillus* strain was able to produce bacteriocins active against *L. monocytogenes, L. innocua, S. aureus*, and *E. coli*. Consequently, the antagonistic activity previously observed was due to other mechanisms, such as the production of organic acids. In fact, the pH values of the lactobacilli supernatants ranged between 3.57 and 3.82 for all the *L. crispatus* strains, excepting for BC3. Even *L. gasseri* BC11, BC12 and BC13 supernatants showed pH values below 4.00 (range 3.68–3.80). *L. gasseri* BC9, BC10, and BC14, and *L. vaginalis* strains showed a lower acidification of the culture medium, with pH values higher than 4.00 (range 4.00–5.07).

### Antibiotic susceptibility of vaginal lactobacilli

The antibiotic susceptibility of the vaginal lactobacilli with respect to a wide spectrum of antibiotics is reported in Table 2. The results evidenced that among all the considered antibiotics, Amoxicillin, Ampicillin, Erythromycin, and Penicillin G showed the highest bactericidal effect. The only exception was represented by *L. vaginalis* BC15 which resulted less susceptible to penicillin G with MIC equal to 16 μg/mL compared to all the other tested lactobacilli. *L. gasseri* BC14, *L. vaginalis* BC15, BC16, and BC17 resulted less susceptible to Vancomycin, and Tetracycline. In general, all the considered lactobacilli, except for *L. crispatus* BC3 and *L. vaginalis* BC17, showed low susceptibility to Ciprofloxacin and Levofloxacin, with MICs greater than 32 μg/mL.

**Table 2:** **Evaluation of minimum inhibitory concentrations (MIC, μg/mL) of various antibiotics against vaginal lactobacilli**.

	**Strain**	**Amoxicillin**	**Ampicillin**	**Ciprofloxacin**	**Clindamycin**	**Erythromycin**	**Gentamycin**	**Levofloxacin**	**Penicillin G**	**Tetracycline**	**Vancomycin**
*L. crispatus*	BC1	0.5	0.75	>32	1.5	0.5	28	>32	0.12	1	0.38
	BC2	0.12	0.09	>32	2	0.5	32	>32	0.03	1.5	2
	BC3	0.18	0.18	1.5	32	0.12	>256	2	0.75	0.38	2
	BC4	0.09	0.25	>32	1	0.38	32	>32	0.9	1	0.5
	BC5	0.5	0.75	>32	0.5	0.18	24	>32	0.18	1	0.38
	BC6	0.5	0.75	>32	0.38	0.25	64	>32	0.12	1.5	0.5
	BC7	0.75	0.75	>32	1.5	0.5	24	>32	0.18	1.5	0.38
	BC8	0.38	0.5	>32	16	0.25	192	>32	0.09	2	1.75
*L. gasseri*	BC9	0.25	0.18	>32	0.38	0.12	48	>32	0.06	1.75	1
	BC10	0.09	0.09	>32	4	0.5	64	>32	0.06	0.5	3
	BC11	0.18	0.12	>32	4	0.5	48	>32	0.03	0.75	3
	BC12	0.03	0.06	>32	4	0.75	16	>32	0.015	0.75	3
	BC13	0.12	0.38	>32	8	0.18	96	>32	0.06	0.75	2
	BC14	0.12	0.18	>32	0.03	0.25	24	>32	0.25	2	128
*L. vaginalis*	BC15	0.5	0.38	>32	3	0.75	8	>32	16	8	128
	BC16	0.25	0.12	>32	3	0.75	4	>32	0.25	4	128
	BC17	0.09	0.12	4	0.03	0.25	4	8	0.25	2	>256

*The MIC values reported are the average of three replicates*.

### Fermentation kinetics of vaginal lactobacilli in pasteurized milk and viability during the refrigerated storage

To assess the technological potential of the vaginal lactobacilli considered in this study, their fermentation kinetics in pasteurized whole milk at 37°C were investigated. The results highlighted the limited technological aptitude of the lactobacilli (Table 3). In fact, none of the strains was able to acidify the milk to pH values below 5.0 in 53 h at 37°C. *L. crispatus* BC4 showed the faster fermentation kinetics reaching pH value of 5.02 in 48 h. Also, a reduced growth capability of the *Lactobacillus* strains in fermented milk was observed (Table 4). The addition of 0.5% of tryptone to the whole pasteurized milk permitted to *L. crispatus* BC1 and BC4, and *L. gasseri* BC12 to reach pH values below 5 within 22 h of incubation at 37°C (Table 5). After 30 h of incubation, also the milks inoculated with *L. crispatus* BC5, BC6, BC7, and BC8 reached pH values below 5.0. Since such fermentation times are not compatible with the industrial protocols for the production of fermented milk, the potential of these *Lactobacillus* strains as adjuncts in dairy product was evaluated, studying their survival during the refrigerated storage of the inoculated milks. The results showed that none of the considered strains was able to grow at the adopted conditions (Table 6). However, some strains, such as *L. crispatus* BC2 and BC3, *L. gasseri* BC9 and BC12, and *L. vaginalis* BC15, showed reduced viability losses compared to the other tested strains. In fact, their viability reductions during the 28-day storage at 4°C was lower than 1.5 log CFU/mL, while the other strains showed a reduction higher than 2 log CFU/mL.

**Table 3:** **Acidification Kinetics (expressed as decrease of pH values) of the vaginal lactobacilli in pasteurized whole milk in relation to the incubation time in hours (t3–t53) at 37°C**.

**Strain**		**t3**	**t6**	**t9**	**t11**	**t14**	**t16**	**t20**	**t24**	**t27**	**t31**	**t34**	**t37**	**t42**	**t48**	**t53**
*L. crispatus*	BC1	6.56	6.51	6.32	6.28	6.10	5.85	5.81	5.77	5.74	5.67	5.58	5.29	5.28	5.17	5.16
	BC2	6.58	6.53	6.44	6.37	6.21	6.13	6.20	6.11	5.99	5.78	5.69	5.61	5.55	5.45	5.38
	BC3	6.61	6.53	6.36	6.30	6.21	6.08	6.13	6.11	6.16	6.15	6.13	6.02	6.14	6.01	5.95
	BC4	6.57	6.40	6.25	6.22	6.02	5.87	5.77	5.60	5.60	5.51	5.44	5.31	5.25	5.02	5.00
	BC5	6.57	6.54	6.44	6.35	6.16	6.04	5.87	5.79	5.76	5.64	5.53	5.30	5.26	5.14	5.13
	BC6	6.56	6.50	6.36	6.31	6.06	5.95	5.79	5.72	5.68	5.59	5.51	5.27	5.25	5.10	5.09
	BC7	6.55	6.49	6.34	6.27	6.10	6.01	5.84	5.67	5.68	5.60	5.50	5.31	5.30	5.13	5.13
	BC8	6.56	6.48	6.39	6.32	6.26	6.25	6.21	6.20	6.25	6.24	6.03	5.91	6.21	6.16	6.28
*L. gasseri*	BC9	6.59	6.53	6.47	6.39	6.36	6.34	6.30	6.24	6.28	6.31	6.24	6.12	6.23	6.15	6.26
	BC10	6.59	6.52	6.41	6.34	6.23	6.21	6.09	6.07	6.05	6.02	6.02	5.76	5.68	5.80	5.83
	BC11	6.55	6.51	6.43	6.36	6.18	6.05	5.89	5.78	5.77	5.63	5.45	5.33	5.26	5.05	5.01
	BC12	6.62	6.54	6.37	6.32	6.23	6.09	6.11	6.07	6.04	6.01	5.97	5.94	5.92	5.88	5.82
	BC13	6.56	6.50	6.39	6.33	6.24	6.13	6.02	5.91	5.84	5.79	5.68	5.45	5.36	5.24	5.19
	BC14	6.63	6.55	6.39	6.31	6.24	6.11	6.07	6.02	5.98	5.94	5.89	5.84	5.77	5.71	5.65
*L. vaginalis*	BC15	6.58	6.41	6.27	6.21	6.04	5.96	5.87	5.79	5.78	5.67	5.55	5.48	5.46	5.38	5.31
	BC16	6.58	6.43	6.40	6.31	6.26	6.19	6.12	6.03	5.98	5.92	5.88	5.81	5.76	5.69	5.61
	BC17	6.55	6.48	6.37	6.28	6.16	6.06	6.01	5.92	5.84	5.77	5.69	5.67	5.51	5.46	5.33

*The pH values are the average of three replicates, the variability for all samples was always <5%*.

**Table 4:** **Microbial viability of vaginal ***Lactobacillus*** strains inoculated in pasteurized whole milk (log CFU/mL ± standard deviation) immediately after inoculation (t0) and after 24 h (t24) of incubation at 37°C**.

		**t0**	**t24**
*L. crispatus*	BC1	6.84 ± 0.23	8.06 ± 0.33
	BC2	6.96 ± 0.18	7.75 ± 0.22
	BC3	6.95 ± 0.21	7.87 ± 0.17
	BC4	6.88 ± 0.33	7.97 ± 0.22
	BC5	7.00 ± 0.18	7.95 ± 0.18
	BC6	7.14 ± 0.26	8.01 ± 0.27
	BC7	6.95 ± 0.31	8.21 ± 0.31
	BC8	6.90 ± 0.24	7.38 ± 0.38
*L. gasseri*	BC9	6.72 ± 0.33	7.79 ± 0.19
	BC10	6.85 ± 0.17	8.05 ± 0.24
	BC11	6.94 ± 0.25	8.23 ± 0.27
	BC12	6.86 ± 0.27	8.02 ± 0.36
	BC13	7.04 ± 0.19	8.11 ± 0.22
	BC14	6.98 ± 0.34	7.86 ± 0.21
*L. vaginalis*	BC15	6.89 ± 0.22	7.98 ± 0.33
	BC16	6.78 ± 0.24	7.75 ± 0.34
	BC17	7.01 ± 0.12	7.97 ± 0.28

*The cell counts are the average of three replicates, the values are reported ± standard deviations (SD)*.

**Table 5:** **Acidification kinetics (expressed as decrease of pH values) of the vaginal lactobacilli in pasteurized whole milk enriched with 0.5% tryptone in relation to the incubation time in hours (t0–t48) at 37°C**.

**Strain**		**t0**	**t4**	**t6**	**t8**	**t10**	**t13**	**t15**	**t17**	**t19**	**t22**	**t24**	**t26**	**t28**	**t30**	**t33**	**t38**	**t40**	**t42**	**t44**	**t48**
*L. crispatus*	BC1	6.70	6.49	6.47	6.43	6.36	5.83	5.62	5.37	5.16	4.94	4.92	4.89	4.78	4.63	4.48	4.14	4.12	4.08	4.05	4.01
	BC2	6.70	6.36	6.37	6.33	6.31	6.25	6.24	6.17	6.12	6.08	6.05	6.03	6.02	5.97	5.93	5.90	5.89	5.86	5.83	5.80
	BC3	6.70	6.35	6.35	6.33	6.33	6.28	6.24	6.25	6.13	6.10	6.08	6.07	6.07	6.10	6.16	6.13	6.14	6.12	6.11	6.10
	BC4	6.70	6.48	6.37	6.21	6.01	5.70	5.52	5.30	5.09	4.82	4.48	4.33	4.21	4.07	4.01	3.98	3.91	3.86	3.82	3.75
	BC5	6.70	6.49	6.48	6.43	6.37	6.04	5.80	5.57	5.34	5.09	4.96	4.82	4.66	4.51	4.35	4.15	4.09	4.03	3.96	3.89
	BC6	6.70	6.44	6.43	6.40	6.33	6.02	5.79	5.56	5.33	5.08	4.99	4.84	4.72	4.56	4.40	4.19	4.09	4.03	3.96	3.91
	BC7	6.70	6.45	6.41	6.29	6.18	5.94	5.77	5.62	5.45	5.27	5.25	5.19	5.09	4.99	4.88	4.66	4.56	4.49	4.42	4.39
	BC8	6.70	6.51	6.44	6.34	6.26	6.23	6.12	5.98	5.84	5.67	5.51	5.43	5.35	4.99	4.97	4.88	4.88	4.87	4.86	4.85
*L. gasseri*	BC9	6.70	6.53	6.50	6.51	6.49	6.41	6.34	6.34	6.29	6.20	6.18	6.17	6.04	5.83	5.65	5.31	5.18	5.04	4.91	4.79
	BC10	6.70	6.47	6.33	6.26	6.18	5.94	5.80	5.68	5.52	5.38	5.33	5.25	5.16	5.09	5.02	4.81	4.78	4.72	4.65	4.62
	BC11	6.70	6.41	6.42	6.36	6.27	6.01	5.94	5.87	5.75	5.67	5.62	5.60	5.57	5.55	5.53	5.52	5.50	5.49	5.42	5.38
	BC12	6.70	6.57	6.53	6.41	6.38	6.11	5.87	5.49	5.12	4.78	4.65	4.55	4.48	4.36	4.32	4.28	4.26	4.23	4.13	4.08
	BC13	6.70	6.54	6.45	6.36	6.27	6.01	5.87	5.76	5.61	5.49	5.41	5.32	5.25	5.18	5.13	5.08	5.07	5.02	4.89	4.84
	BC14	6.70	6.65	6.65	6.65	6.65	6.59	6.58	6.54	6.50	6.46	6.45	6.53	6.52	6.50	6.50	6.50	6.49	6.35	6.25	5.79
*L. vaginalis*	BC15	6.70	6.63	6.63	6.63	6.63	6.54	6.45	6.38	6.32	6.30	6.18	6.12	6.08	6.01	5.95	5.93	5.92	5.91	5.88	5.81
	BC16	6.70	6.65	6.64	6.63	6.63	6.56	6.52	6.46	6.41	6.38	6.29	6.21	6.18	6.11	6.07	6.03	6.01	5.98	5.95	5.91
	BC17	6.70	6.63	6.63	6.63	6.63	6.54	6.53	6.49	6.46	6.41	6.40	6.40	6.38	6.37	6.36	6.35	6.30	6.29	6.20	6.05

*The pH values are the average of three replicates, the variability for all samples was always <5%*.

**Table 6:** **Microbial viability of vaginal ***Lactobacillus*** strains inoculated in pasteurized whole milk (log CFU/mL ± standard deviation) immediately after inoculation (t0) and after 7 (t7), 14 (t14), 21 (t21), and 28 (t28) days of refrigerated storage at 4°C**.

**Strain**		**t0**	**t7**	**t14**	**t21**	**t28**
*L. crispatus*	BC1	6.93 ± 0.22	6.79 ± 0.22	6.31 ± 0.31	6.20 ± 0.28	5.77 ± 0.31
	BC2	7.91 ± 0.33	7.60 ± 0.39	7.11 ± 0.39	7.27 ± 0.34	6.47 ± 0.28
	BC3	8.13 ± 0.35	7.89 ± 0.33	7.92 ± 0.41	7.90 ± 0.41	7.82 ± 0.34
	BC4	6.78 ± 0.21	5.54 ± 0.41	6.40 ± 0.24	6.29 ± 0.22	5.96 ± 0.18
	BC5	6.60 ± 0.19	6.25 ± 0.31	5.63 ± 0.19	5.45 ± 0.36	4.75 ± 0.39
	BC6	6.78 ± 0.28	6.19 ± 0.28	6.02 ± 0.25	5.81 ± 0.29	5.13 ± 0.41
	BC7	7.23 ± 0.33	6.57 ± 0.33	6.15 ± 0.33	6.13 ± 0.31	5.68 ± 0.34
	BC8	7.48 ± 0.31	3.90 ± 0.59	3.59 ± 0.24	2.78 ± 0.27	1.60 ± 0.69
*L. gasseri*	BC9	7.18 ± 0.22	6.62 ± 0.29	6.58 ± 0.26	6.01 ± 0.36	6.10 ± 0.27
	BC10	7.10 ± 0.28	6.27 ± 0.34	5.95 ± 0.33	5.36 ± 0.35	5.07 ± 028
	BC11	7.20 ± 0.39	6.19 ± 0.24	5.82 ± 0.24	5.40 ± 0.29	5.18 ± 0.31
	BC12	7.67 ± 0.24	6.92 ± 0.31	6.85 ± 0.19	6.57 ± 0.31	6.26 ± 0.41
	BC13	7.00 ± 0.19	3.24 ± 0.36	3.16 ± 0.48	2.93 ± 0.47	2.43 ± 0.58
	BC14	7.15 ± 0.22	5.51 ± 0.34	4.30 ± 0.54	3.81 ± 0.45	1.48 ± 0.38
*L. vaginalis*	BC15	7.23 ± 0.36	6.84 ± 0.19	7.02 ± 0.28	6.74 ± 0.24	6.44 ± 0.28
	BC16	7.28 ± 0.45	6.59 ± 0.38	6.29 ± 0.25	5.79 ± 0.38	5.34 ± 0.45
	BC17	7.30 ± 0.21	7.04 ± 0.22	6.54 ± 0.17	5.28 ± 0.37	5.02 ± 0.33

*The cell counts are the average of three replicates, the values are reported ± standard deviations (SD)*.

### Volatile molecule profiles of vaginal lactobacilli in pasteurized milk

The analysis of the volatile molecule profiles of milks inoculated with the vaginal lactobacilli and collected after 48 h of incubation at 37°C evidenced the presence of molecules belonging to the chemical classes of acids, ketones and alcohols. The relative percentages of the detected molecules in relation to the strain considered are reported in Table 7. The strains belonging to *L. crispatus* and in a minor extent those belonging to *L. gasseri* resulted characterized by high amount of acetic acid. The strains belonging to *L. crispatus* showed relative percentages of acetic acid ranging between 24.9 and 72.6%, while in case of *L. gasseri* acetic acid ranged between 9.8 and 45.2%. The strains belonging to *L. vaginalis* produced a limited amount of acids, including acetic acid. In fact, relative percentages of acids ranged between 1.9 and 4.0%. The *L. vaginalis* strains showed the highest relative percentages of ethanol, above 50% in case of BC16 and BC17 strains, and 20.5% for BC15. *L. gasseri* BC14 showed a relative percentage of ethanol of 27.2% while all the other *L. gasseri* strains showed relative percentages lower than 10%. Regarding *L. crispatus* species, BC2 and BC3 strains were the highest producers of ethanol, registering values of 24.5 and 16.6%, respectively. The relative percentages of ketones resulted extremely variables in relation to the strain inoculated. Only *L. vaginalis* BC15 showed high relative percentages of 2-butanone and acetoin. Contrarily, the strains belonging to *L gasseri* showed high levels of cyclopentanone and 2-heptanone. Finally, *L. crispatus* BC2 and BC3 evidenced the highest levels of acetone. The presence of diacetyl was detected for all the considered *L. vaginalis* strains and the strains BC4 and BC9. High relative percentages of acetoin were detected for the strains *L. vaginalis* BC15 and *L. gasseri* BC9, BC13 and BC14. However, to a minor extend, the presence of acetoin was detected also for the strains of *L. crispatus* BC1, BC2, BC4, BC7, and BC8. Acetaldehyde was produced by the tested strains in a range between 0.5 and 5.0 % except for the strains *L. vaginalis* BC15-BC17 and the strains BC3, BC6, and BC14.

**Table 7:** **Volatile compounds (expressed as relative percentages) detected through GC-MS-SPME technique in pasteurized whole milk enriched with 0.5% tryptone inoculated with the vaginal lactobacilli after an incubation period of 48 h at 37°C**.

	***L. crispatus***								***L. gasseri***						***L. vaginalis***		
	**BC1**	**BC2**	**BC3**	**BC4**	**BC5**	**BC6**	**BC7**	**BC8**	**BC9**	**BC10**	**BC11**	**BC12**	**BC13**	**BC14**	**BC15**	**BC16**	**BC17**
Acetone	3.6	11.1	11.2	2.5	6.9	7.3	7.9	7.3	4.1	10.3	7.7	5.9	8.0	5.3	0.8	1.2	0.7
2-Butanone	0.4	2.1	2.5	0.4	0.6	0.9	1.4	1.0	1.9	2.7	1.5	1.6	1.4	1.0	29.4	0.9	0.3
Diacetyl	0.0	0.0	0.0	0.2	0.0	0.0	0.0	0.0	3.6	0.0	0.0	0.0	0.0	0.0	0.7	1.7	1.6
2-Pentanone	0.8	3.5	3.0	1.1	1.3	1.7	3.1	1.7	4.2	4.2	3.3	5.3	10.0	4.4	1.9	2.9	1.7
Isobutenyl ketone-	1.2	1.6	1.1	2.0	1.8	0.8	1.3	2.7	7.8	3.3	2.2	1.8	3.9	1.6	0.2	1.0	1.4
2-Heptanone	5.3	10.3	9.3	4.3	7.1	10.0	6.9	4.3	11.5	12.2	8.1	9.9	11.0	6.5	4.5	1.9	1.4
2-Hexanone, 5-methyl-	0.0	0.0	0.0	0.0	0.0	1.1	1.2	0.0	0.0	2.0	0.0	0.9	6.1	3.2	0.4	0.0	0.0
Dihydro-2-methyl-3(2H)-furanone	0.0	0.0	0.0	0.0	0.0	0.4	0.0	0.4	0.0	0.0	1.7	3.5	2.8	0.5	0.0	0.0	0.0
Acetoin	1.2	0.9	0.0	2.6	0.0	0.0	1.7	0.2	4.4	0.0	0.0	0.0	6.6	4.6	18.4	0.0	0.0
2-Nonanone	0.4	0.0	0.0	0.8	1.1	0.0	0.0	0.0	0.0	0.0	0.0	0.0	0.0	0.0	0.0	0.0	0.0
Cyclopentanone, 3-(3-hydroxy-1-propenyl)-)-	4.0	10.9	14.1	6.0	1.4	7.4	8.6	11.9	22.9	10.3	9.7	14.2	23.8	19.3	5.3	21.8	26.6
Total ketones	16.9	40.3	41.2	20.0	20.2	29.5	32.0	29.5	60.5	45.0	34.2	43.0	73.5	46.3	61.5	31.5	33.7
Ethyl Acetate	0.0	0.5	2.8	0.0	0.0	0.0	0.3	0.0	0.0	0.1	0.0	0.0	0.0	0.0	0.2	0.4	0.5
Butanoic acid, ethyl ester	0.0	0.5	1.4	0.0	0.0	0.0	0.1	0.0	0.0	0.0	0.0	0.0	0.0	0.0	0.5	1.6	1.8
Carbonic acid, heptadecyl propyl ester	0.3	1.7	1.4	0.3	0.0	0.0	0.3	0.5	0.9	0.0	0.0	0.6	0.0	1.6	0.7	2.4	2.5
Total esters	0.3	2.7	5.6	0.3	0.0	0.0	0.7	0.5	0.9	0.1	0.0	0.6	0.0	1.6	1.4	4.5	4.9
3-Methylbutanal -	0.0	0.3	0.0	0.0	0.0	0.1	0.1	0.0	0.0	0.0	0.0	0.0	0.0	0.0	1.4	1.2	0.5
Octanal	1.2	0.0	0.0	1.0	0.9	0.0	0.0	2.3	2.8	0.0	0.0	1.0	0.0	0.0	0.0	0.0	0.0
Acetaldehyde	0.6	1.1	0.0	0.5	1.4	0.0	2.3	3.0	3.5	3.9	2.0	1.3	5.0	0.0	0.0	0.0	0.0
Total aldehydes	1.8	1.4	0.0	1.5	2.3	0.1	2.4	5.3	6.3	3.9	2.0	2.4	5.0	0.0	1.4	1.2	0.5
Ethanol	0.0	24.5	16.6	0.0	0.0	0.0	5.9	0.0	0.0	0.0	0.0	8.9	1.0	27.2	20.5	51.0	51.2
2,7-Dimethyl-1-octanol -	1.5	0.8	1.5	0.6	0.4	0.5	0.6	2.1	2.3	1.5	1.1	1.5	2.3	1.7	0.5	1.6	2.0
3-Methyl-1-butanol -	0.0	0.0	0.0	0.0	0.0	0.0	0.0	0.0	0.0	0.0	0.0	0.0	0.0	6.4	10.0	1.0	0.4
6-Methyl-2-heptanol	0.2	0.2	0.0	0.4	0.4	0.3	0.2	0.5	1.6	0.9	0.6	0.4	1.0	0.3	0.1	0.3	0.4
Total alcohols	1.7	25.5	18.1	0.9	0.8	0.8	6.7	2.6	3.9	2.4	1.7	10.8	4.3	35.5	31.0	53.9	54.0
Acetic acid	72.6	24.9	31.6	68.2	67.7	62.0	48.5	53.8	17.8	29.6	45.2	30.0	9.8	10.3	2.4	2.6	0.0
Butanoic acid	2.5	0.8	0.0	2.8	2.2	2.5	1.8	1.9	0.4	5.3	5.3	3.9	1.4	1.1	0.3	1.5	1.9
Hexanoic acid	1.8	0.0	0.0	3.2	3.5	3.7	4.9	2.8	3.9	8.5	7.7	7.3	0.0	0.0	0.0	0.0	0.0
Total acids	76.9	25.7	31.6	74.3	73.5	68.2	55.2	58.5	22.1	43.3	58.3	41.2	11.2	11.4	2.7	4.0	1.9
Total compounds	97.5	95.6	96.5	97.0	96.8	98.5	97.0	96.3	93.7	94.7	96.1	98.0	94.0	94.8	98.0	95.1	94.9

*The results are means of three independent experiments. The coefficients of variability, expressed as the percentage ratios between the standard deviations and the mean values, ranged between 2 and 10%*.

## Discussion

The administration of probiotics has been shown to be effective in restoring a normal vaginal and gut microbiota, contributing to resolve gynecological disorders (Borges et al., 2014). The rationale behind the present work is to develop a functional food for the female gender capable of delivering probiotic lactobacilli in the vagina in order to promote the gynecological health through nutrition. Taking an active ingredient through a food is a form of administration that best meets the compliance of the consumer and this aspect represents an important added value of a functional food. In particular, the anti-*Candida* and antibacterial activities of the *Lactobacillus* strains used in this work can be exploited to restore the healthy status of the human vaginal and gut microbiota, contributing to protect the woman from vaginal dysbiosis and infections. However, the formulation of a functional dairy product should be subordinate to the selection of probiotic strains able to maintain viability and functionality during storage and without negative effects on the hedonistic properties of the carrier food. In fact, to exert positive health effects, the functional microorganisms need to be viable, active, and sufficiently abundant, in concentrations of at least 6 log CFU/g throughout the shelf life, and consequently they have to maintain their viability during the refrigerated storage (Tripathi and Giri, 2014; Burns et al., 2015).

Our data clearly indicated the unsuitability of the studied strains as fermentation starters for the industrial production of dairy products. In fact, the fermentation kinetics in pasteurized whole milk have shown that none of the strains could acidify the milk to pH values below 5 in a considered time of 53 h at the temperature of 37°C. Even the addition of 0.5% tryptone, to provide a source of protein and promote the growth kinetics of the tested strains, did not increase the acidification rate to acceptable levels for the dairy industries. Nevertheless, some *Lactobacillus* strains maintained a high viability for over a month in pasteurized whole milk at 4°C, indicating their suitability in dairy products as adjunct cultures instead of starters. This is a very important result from an applicative point of view: the use of vaginal lactobacilli as additional cultures in fermented milks and fresh cheeses, which represent the most used carriers for functional microorganisms, allows to overcome the typical sensory defects of the products obtained using probiotics as starters (Castro et al., 2015). Similarly, Patrignani et al. (2006) reported, for the functional strains belonging to the species *L. gasseri, L. plantarum, L. rhamnosus* isolated from Masaai milks, slow fermentation kinetics, not acceptable for industrial fermentation. In sight of this, the probiotic bacteria belonging to *Bifidobacterium* spp. and the species *L. acidophilus, L. casei*, and *L. rhamnosus* are generally used as additional cultures due to their poor fermentation kinetics and the scarce sensory properties of fermented milk obtained when they are used as starters (Patrignani et al., 2007; Settanni and Moschetti, 2010; Castro et al., 2015).

The data reported in this work showed that some *Lactobacillus* strains could maintain a high viability for over a month in pasteurized whole milk at 4°C, indicating their suitability as adjunct cultures in dairy products. This is a very important result from an applicative point of view: the use of vaginal lactobacilli as additional cultures in dairy products, such as fermented milks and fresh cheeses, which represent the most used carriers for functional microorganisms, allows to correct the typical sensory defects of the products obtained using probiotics as starters (Castro et al., 2015). On the other hand, to produce a satisfactory fermented probiotic milk product, the viable cell count at the time of consumption should be above 6 log CFU/g in order to comply with the International Dairy Federation standards (IDF 1992) and to ensure the supply of a sufficient “daily dose” of viable bacteria (Patrignani et al., 2006, 2016). Consequently, the most important criterion to select probiotic strains to be employed in fermented milk products as adjuncts, is their ability to maintain high viability during manufacture and refrigerated storage (Kailasapathy and Chin, 2000; Soccol et al., 2010). In fact, several cases of marked loss of viability of the most common probiotics belonging to *Bifidobacterium* spp. and *L. acidophilus* species in refrigerated dairy products have been reported in literature (Patrignani et al., 2006).

In this work, we demonstrated that several vaginal lactobacilli, mainly belonging to *L. crispatus* species, exhibited a remarkable antagonistic activity against spoilage and pathogenic microorganisms of food interest, as well as against urogenital pathogens. In addition, previous literature data had clearly indicated that the vaginal lactobacilli considered in this study, especially *L. crispatus* strains, were characterized by significant anti-*Candida* (Parolin et al., 2015) and anti*-Chlamydia* (Nardini et al., 2016) activities. The results obtained in this research showed the absence of bacteriocin production by all the tested strains. The highest antagonistic activities of *L. crispatus* BC1, BC4, BC5, and BC6 can be attributed to the highest production of organic acids as demonstrated by the pH values of the cell free supernatants, and the volatile molecule profiles of the inoculated milks registering the highest abundances of acetic acid and short chain fatty acids (hexanoic and butanoic acid). The high levels of acetic acid detected in the volatile molecule profiles of the *L. crispatus* strains can explain their high antimicrobial activities against the target microorganisms. In fact, it is well reported that short-chain organic acids, such as acetic acid and lactic acid, can exert a strong antimicrobial activity against both gram-positive and gram-negative microorganisms, due to the diffusion of the acids through the cell membrane of microorganisms (Varalakshmi et al., 2014; Poppi et al., 2015). Also Annuk et al. (2003) attributed the strong antimicrobial activity of intestinal lactobacilli against both Gram-negative and Gram-positive bacteria to the high production of organic acids, such as lactic acid, succinic acid and acetic acid. Moreover, Breshears et al. (2015) showed that high concentrations of acetic acid could inhibit the growth of *Neisseria gonorrhoeae* on porcine vaginal mucosal model at pH 5.5. In addition, all the tested lactobacilli showed the production of diacetyl, acetoin, and also acetaldehyde, which are important for the sensory characterization of dairy products as well as for their antimicrobial activity against both gram-positive and gram-negative pathogenic species (Ott et al., 1997; Lanciotti et al., 2003; Patrignani et al., 2007, 2016). Such molecules are considered key factor for the characterization of dairy products (Escamilla-Hurtado et al., 2000; Lanciotti et al., 2003, 2007). Furthermore, the capacity to generate diacetyl and acetoin is considered a general trait relevant for the selection of starter lactic acid bacteria (Cruciata et al., 2014). Moreover, acetaldehyde is considered a fundamental compound for fermented milks quality as it gives a characteristic flavor in yogurt and fermented milks (Smit et al., 2005; Patrignani et al., 2007). These results are particularly important since the ability to impart good sensory properties is an important selection criterion for starter cultures or probiotic adjuncts (Patrignani et al., 2006; Cheng, 2010; Routray and Mishra, 2011; Widyastuti and Febrisiantosa, 2014).

The determination of the antibiogram is considered a prerequisite in protocols for the selection of starter, co-starter or functional microorganisms by EFSA (Wedajo, 2015).

The antibiogram results are in agreement with literature data (Ammor et al., 2007; Nueno-Palop and Narbad, 2011; Fguiri et al., 2015). In fact, the lactobacilli showed a variable spectrum of susceptibility in relation to the species and the strain considered. The majority of the tested strains resulted very sensitive to Amoxicillin, Ampicillin, Penicillin G and Erythromycin. Some lactobacilli were found to be less susceptible to different antibiotics. In these case, further studies are needed to better characterize the resistance mechanism, before including these strains in food products. Other authors have observed a high susceptibility of different lactic acid bacteria strains to these antibiotics (Danielsen and Wind, 2003; Temmerman et al., 2003; Nueno-Palop and Narbad, 2011). In general, the spectrum of susceptibility to antibiotics results variable in function of the lactic acid bacteria strain considered. In fact, several studies have shown the natural resistance of lactic acid bacteria to a wide range of antibiotics (Botes et al., 2008; Fguiri et al., 2015).

Although strains belonging to *L. casei* (*L. paracasei*), *L. rhamnosus*, and *L. plantarum* are widely used in probiotic dairy products (Kaushik et al., 2009; Zheng et al., 2013), only few reports are focused simultaneously on their functional and technological properties (Olasupo et al., 2001; Patrignani et al., 2006). Some aspects, i.e., viability in milk during refrigerated storage, antagonistic activity against pathogenic and spoilage species, volatile molecule profiles and production of key sensory compounds such as dyacetyl, are particularly important for the selection of adjunct cultures to produce dairy functional foods since they are the main choice criteria for the consumers and the dairy industries. *L. crispatus* BC1, BC2, BC3, BC4, and BC5, *L*. *gasseri* BC9 and BC12, and *L. vaginalis* BC15 maintained a high vitality during refrigerated storage and at the same time they showed a good antimicrobial activity against both spoilage and pathogenic microorganisms. In addition, they showed interesting volatile molecule profiles, without negative aroma compounds for dairy products. These strains, with proven anti-*Candida* and antibacterial activities, appear to be the most promising candidates to be used as adjunct cultures for the production of a functional food capable of improving the health of the female gender.

## Conclusions

The results obtained represent a first towel for the selection of adjunct cultures to produce new functional dairy products, directed to the women well-being, contributing also to innovate the dairy sector. In fact, in recent years there has been a significant increasing demand for tailor made microbial strains able to contribute to food innovation in terms of functionality, sensory, nutritional and rheological characteristics. The present study has identified a pool of strains having technological and functional properties promising for the production of a dairy functional food specifically directed to female gender.

## Author contributions

All the authors contributed to the experimental design of the work; as well as to the acquisition, analysis and interpretation of the obtained results; Moreover, all the authors contributed to the writing and the critical revision of the manuscript.

### Conflict of interest statement

The authors declare that the research was conducted in the absence of any commercial or financial relationships that could be construed as a potential conflict of interest.
